# An introduction to process tracing as an innovative qualitative research method to explore affective variables in SLA

**DOI:** 10.3389/fpsyg.2022.984444

**Published:** 2023-01-06

**Authors:** Wenxiao Yan

**Affiliations:** School of Foreign Languages, Henan Institute of Science and Technology, Xinxiang, China

**Keywords:** process tracing, L2 affective variables, complex dynamic systems theory, qualitative research, SLA

## Abstract

When the Complex Dynamic Systems Theory (CDST) enlightened the line of inquiry in education, innovative research methodologies, both quantitative and qualitative, were also introduced. Process tracing, which is among the CDST-compatible qualitative research methods, has just begun to benefit SLA research in the past few years. The present study provides a review of the conceptualization, significance, and procedural features for the implementation of the process tracing analytical method. In doing so, this review suggests a number of practices through which process tracing has been introduced in SLA. Additionally, some practical implications are provided for SLA researchers to enhance their knowledge of this new approach. Finally, future research suggestions for a more advanced use of this method are made in SLA.

## Introduction

In recent years, academic and scientific research domains have been significantly enriched with the complex dynamic systems theory (CDST), which has opened up new horizons to describe the structure of human social life qualitatively and quantitatively. In the present study, we point out how CDST has also informed language studies, and why it seems crucial to develop or use innovative research methodologies that can appropriately manifest the dynamicity at the heart of the language development process that takes place within a classroom learning context. Then, we go on with the introduction of process tracing as an innovational approach to qualitative research, which is fit for tracing the language learning process, dynamically set in a network of conjoined reciprocal forces in class. Finally and most importantly, we review the existing body of L2 studies that have benefited from this analytical method so far.

We come to the conclusion that although these studies have been indeed very limited in number, they have managed to significantly contribute to the SLA theory and practice. We will review the significance of these studies and the potential implications they can have for the L2 teaching and learning domain. Becoming familiar with the procedural details of these studies which employed the process tracing approach can direct future lines of L2 learning/teaching research. We will review how this innovative research method, which follows a qualitative approach to research can be effectively used in exploratory as well as causal works of research into L2 development. More specifically, we will see how this analytical method suits the investigation of affective variables involved in language learning, which have a developmental nature. Process tracing can show the nuances of how these constructs develop during the classroom-embedded language learning experience.

## Review of the main concepts

### Dynamic phase in SLA research

Language learning lies within the more general realm of education, yet the domain-specific body of SLA research has prevailed for decades. The dynamic phase has recently begun and addressed different aspects of language learning and teaching. For several reasons, CDST can be considered perfectly suited for language studies. As it stands against the deterministic predictability of dynamic phenomena and resists the conventional approaches to language development, and it takes into account the continuous effects of time and place on the language learning process, CDST suits the language-related body of research (Hiver and Al-Hoorie, 2020). The need to explore language learning constraints from a dynamic approach, consider the variability in intra- and inter-individual emotions or behaviors and study the processes of change in the reality of classroom learning, was first pinpointed by Larsen-Freeman (1997). SLA-related phenomena are marked by inter-disciplinary and openness to external influences, which makes CDST an appropriate framework for studies in this area. A fully informative overview of CDST in applied linguistics was published by Larsen-Freeman and Cameron (2008), raising awareness of the self-organizing, interconnected and co-adaptive quality of language learning and approaching language process as always emerging from interactions.

Attempts to systematically study the processes of change in language development and bring substantial evidence for that require efficient methodological procedures. According to Hiver and Al-Hoorie (2020), SLA researchers need to go beyond describing and theorizing changes in dynamic language systems and should proceed with the gathering of evidence for language development or the growth of specific learner/teacher-related emotions or behaviors based on the CDST. Yet, many researchers in SLA fall behind the use of research methodologies that can sufficiently represent inter- and intra-individual differences along with the spatiotemporal limits of language learning due to the ineffective and, better say, inefficient research methods they employed to trace the processes of change in the development of any language-related variable. I move on to introduce process tracing as an effective research methodology in qualitative language studies. Then, the existing body of SLA research that used this innovative research method is reviewed to see what valuable contributions they managed to make to the field of SLA theory and practice.

### Distinctive features of process tracing

The literature of the method indicates three types of process tracing: (a) theory testing process tracing, (b) theory building process tracing, and (c) case-centric process tracing. The difference between the first two types and the latter is that the first two types focus on the development of a generalizable theory but the case-centric type aims to develop a theory relevant to a particular case under investigation. Thus, the first two types of process tracing are used as quantitative methods but the case-centric one is used as a qualitative method. Case-centric process-tracing refers to a within-case method (i.e., a particular kind of case study) to unravel complex causal underpinnings on a micro-scale of analysis (Checkel, 2007). It hopes to infer the causal underpinnings of that case, the dynamic development and its emanant outcomes (Mahoney, 2012, 2015). The process tracing approach has proved successful in unwrapping the black box of the cause-and-effect relations underlying a particular variable. The kind of process-tracing mostly used in the SLA body of research is case-centric, which refers to the unpacking of the causes of a particular outcome in only one case and is of a higher benefit to qualitative researchers (Beach and Pedersen, 2013). This is contained among the more inductive kinds of process tracing, according to Trampusch and Palier (2016). Nearly all inductive kinds of process tracing see time as a major variable involved in the causal justification of a certain behavior, emotion, personality trait and so on. And they mostly base their explanatory analysis on the different existing theoretical explanations to explain the temporally stepwise procedure of a certain condition (Trampusch and Palier, 2016).

It should be noted that the dynamic turn in the field of SLA has been quite recent and; thus, the generation of theories with regard to specific cases that cannot be generalized to other contexts has been needed by the researchers in this field. The introduction of case-centric process tracing is a response to this need. The word “tracing” in process tracing refers to the tracing of evidence based on which proof for the hypothetical causal mechanisms can be given. Following the procedures put forth by Bennett and Checkel (2015), the first step of conducting a process training in SLA studies is to specify the variable as the target outcome in the experience of learning the foreign or second language. The aim is to trace the causal mechanisms underlying the process of learning L2 naturally in an EFL course. The second step is to select a proper case, who is taking up the language course at a prespecified proficiency level, and who is wholeheartedly willing to take part in the study and give the required information in the different steps of the language program. In the third step, to avoid confirmation bias, it should be attempted to take into account all primary alternative explanations for the variable of interest (e.g., boredom, enjoyment, etc.) including the different models and theories underlying the causes of the variable. To do without the cognitive bias of seeing patterns where there was none, as recommended by George and McKeown (1985), the researchers should specify theoretically sound causal path alternatives sooner in order to do the process verification.

As the fourth step, the alternative explanations are collected from the literature assigned to and two broad categories: theoretical or non-theoretical. The former includes the existing theories that deal with the causes of the variable. The latter involves the other factors not addressed in the relevant theories of the variable of interest but found in different works of research and presented as the probable causes of the variable by several researchers. Examples are disengagement (Fahlman, 2009; Pekrun et al., 2010), class time, the total load of work, inadequate feedback, lack of congruity between class-related tasks and L2 proficiency, teacher’s use of language learning grouping, subjects to cover (Chapman, 2013; Kruk and Zawodniak, 2017, 2018) and perceptions toward the teacher (Chapman, 2013).

In the fifth step, the evidence is typically accumulated from the journal writings and from answers provided to interview questions phase by phase and probably session by session. As the sixth step, the explanations (theoretical or non-theoretical) which are not documented in that certain case are discarded and the array of different reasons is narrowed down more up to the final stage of process tracing. And in the seventh step, as described by Bennett and Checkel (2015), when the process goes on smoothly in the next steps, efforts are put to collect more nuanced evidence for the causes that appear to be true more than others, and eventually the tracing stops by the end of the last phase or the last session when the class ends as scheduled.

In the data analysis, typically two or more researchers cooperate in the analysis through the process tracing separately to code the data during first-attempt content analysis. The recurrent themes pointing to the causes of the variable of interest that the case has experienced are highlighted and then organized as the theoretical and non-theoretical categories. The distribution of these categories is to be traced within the whole program. Through the process-tracing, justifications (causal procedures) that are not proven or documented are left out and, thus, the list of alternative causes is narrowed down more. Also, as recommended by Bennett and Checkel (2015), once the process slowly enters the later steps, the researchers try to achieve more in-depth evidence for reasons that have turned out to be true more frequently than others.

### Process tracing and a complex dynamic systems theory approach to study L2 learners’ affective variables

The SLA-related literature so far has drawn attention to several affective variables playing a role in the process of L2 learning. Instances are learners’ self-confidence (de Saint Léger and Storch, 2009; Peng and Woodrow, 2010; Lee and Lee, 2019), anxiety involved in language learning (Macintyre and Legatto, 2011; Lee, 2019; Lee and Lee, 2019), L2 motivation (MacIntyre et al., 2002; Yu, 2011; Khajavy et al., 2016; Lee, 2019; Lee and Lee, 2019; Pishghadam et al., 2021), grit (Duckworth et al., 2007; Lee and Lee, 2019), and boredom (Pekrun et al., 2010; Goetz et al., 2014; Derakhshan et al., 2021a,b; Pawlak et al., 2021). Though these affective variables have been in the interest of SLA researchers for years, studies of these affective variables based on the CDST are still limited.

L2 affective constructs of interest in the recent positive psychology movement in SLA, such as both positive and negative emotions, has been susceptible to the dynamic turn in this field. This means that they have been attempted to be investigated as states which can change under the influence of situational factors. Based on the integration of these two movements, the need for the development of case-centric theories which are sensitive to a given context has been felt more than before. As contended by Dewaele and Li (2020), SLA has entered the third phase (i.e., the dynamic phase) of investigating affective variables, coming next to the general and domain-specific phases. This dynamic shift shows concerns about both positive affective variables (e.g., motivation, self-confidence, grit, foreign language enjoyment) and negative constructs (e.g., demotivation, anxiety, boredom). There has been a shift in SLA research to examine the development of learner-related affective variables by tracing their complex dynamic interactions.

It is fortunate that more CDST-compatible research designs have emerged in the past few years to investigate L2 learners’ affective variables. Among the innovative qualitative methods have been process tracing, ecological assessment and social network analysis. Process tracing seems to have managed to produce more fruitful findings as it proved to be a robust systematic qualitative approach to exploring causal relations. There are reasons why it is thought that process tracing should effectively be used to explore language learners’ affective variables. The recent literature on L2 learners’ affective variables guided by the CDST showed that all learners’ emotions either positive or negative are dynamically formed within the network of class learning, in which a number of actors are involved including teacher- and learner-related variables and variables related to the learning environment. As a result of the dynamic turn in the field of SLA, L2 affective variables should be seen as patterns which self-organize in different states. This self-organization takes place under the influence of several causal mechanisms whose explanations need evidence. Case-centric process tracing provides the tracing of this evidence as proof for the existence of the hypothesized causal mechanisms. Besides, as contended by Joe et al. (2017), in a safe, positive, and highly supportive classroom context, reciprocal attention and productive communications and social interactions between the human actors are encouraged best. Within such a classroom environment, learning is combined with joy and strengthened interpersonal affairs; thus, positive affective variables can be enhanced (Dewaele and MacIntyre, 2014). Admittedly, teachers play a major role in creating such a caring class environment to increase positive affects increase the level of self-confidence, motivation, enjoyment and decrease in boredom in the classroom, as evidenced by a number of studies (e.g., Dewaele and MacIntyre, 2014; Khajavy et al., 2018; Dewaele et al., 2019; Derakhshan et al., 2021a; Elahi Shirvan and Taherian, 2021). Process tracing can hopefully consider this network of interacting forces in forming and growing language learners’ affective variables, and can effectively be used to model the casual paths of language learners’ growing emotions. Case-centric process tracing has been also a response to the ergodicity issue concerning the research on individual differences in SLA, especially when it comes to the investigation of L2 affective variables. This means that since the behavior or emotion of each individual language learner is unique and does not necessarily represent the average, language learners can hardly be considered as ergodic ensembles (see Lowie and Verspoor, 2019). This ergodicity issue requires the use of appropriate methods for tracing how individual patterns of L2 affective variables can be realized in different situations. One of these methods is case-centric process tracing. This means that the use of the case-centric approach does not follow a nomothetic view in terms of the generation of generalizable theory to be built or tested with regard to L2 affective variables, Instead, it holds an idiographic perspective in which the theories developed out of the investigation of L2 affective variables are limited to a specific case and are not generalizable to other contexts. It is worth noting that case-centric process tracing deals with a deep investigation of a single case. This case in the investigation of L2 affective variables can be a single individual. Thus, one individual can be the main focus of a given study *via* case-centric process tracing. The findings of studies using a process tracing approach to the exploration of the causal traces underlying L2 learners’ affective factors can inform L2 teachers of these variables and encourage them to find ways to positively influence learners’ emotions.

### Review of process tracing in SLA research

The studies in L2 education domain which have used the process tracing are limited in number, and they have been conducted within the past 2 years. Yet, they had a significant contribution to the field, as will be reviewed here. A summary of these published works of research are included in Table 1.

**TABLE 1:** A summary of the related literature on process tracing in language studies.

Title of paper	References	Journal	Purpose of study	Main findings
Challenges of online language teaching during the COVID-19 pandemic: A process tracing approach	Shahnama et al., 2021	Journal of Teaching Language Skills	to excavate the challenges faced by an EFL teacher during an online EFL course at intermediate level of proficiency	The lack of technological resources posed the greatest problems during the course.
A process tracing study of the dynamic patterns of boredom in an online L3 course of German during COVID-19 pandemic	Yazdanmehr et al., 2021	Foreign Language Annals	to investigate the causal underpinnings of boredom in an online language learning experience	Under-stimulation, limited sense of control of activities and attention deficit showed to be the most important causes of boredom.

The process tracing study conducted by Shahnama et al. (2021) was inspired by the sudden emergence of online language learning on a global scale at the time of the COVID-19 pandemic. The researchers adopted a process-tracing method to the exploration of the causal underpinnings of boredom students felt in an online L3 course. They conducted a case study of an adult learner of German (as L3) and closely examined the descriptions of the boredom she experienced during an entire semester which took 13 sessions long. The trajectory of boredom all over the semester revealed that the student experienced the highest intensity of boredom at the beginning of the course though this perceived boredom continued up to the end of the program. Out of the theoretical causes of boredom, under-stimulation, low perceived control of tasks and attention deficit showed to be the most prevalent up to the end of the program. Out of the non-theoretical causes of boredom, the user-unfriendly needs of online experience were met mostly in the outset of the semester and continued up to the middle yet were found to be less effective in the end of the program. The process tracing method proved efficient in unwrapping the black box of the causal traces leading to boredom in the online L3 learning context. The authors made a number of suggestions as how to predict these sources of boredom and try to make up for the existing boredom in an online L2/L3 program. Suggestions were made on how to make the class more interactive, which seems still essential as the use of online learning is still around more than any time before after the experience of the COVID-19 pandemic.

In another study in the same year, Yazdanmehr et al. (2021) were inspired by the unprecedented prevalence of online education in the post-COVID-19 pandemic worldwide. They decided to investigate the challenges that an English language teacher faced during an online language program. To explore these challenges, they adopted a process-tracing method to dig out the causal traces underlying the challenges in the outset, middle and end of the language program. The research findings revealed that the hardest challenges during the program particularly in the sessions at the beginning and middle of the semester were induced by the lack of technological facilities. The two other causal mechanisms, human and content facilities, were the highest at the beginning of the course but decreased significantly toward the end. The most problematic challenges facing the teacher were restrictions of the platform, internet coverage and the unpreparedness of human actors for the new online mode of learning. The results showed an increase in the teacher’s and also most language learners’ knowledge of technology and media literacy until the end of the course. Yet, the teacher was challenged with several students’ very slow rate of matching with the new mode until the very end of the class. Yazdanmehr et al. (2021) provided some conclusive remarks on how to prevent these challenges or handle them efficiently when they occurred, especially in developing countries, with many limited infrastructures needed for online education.

### Insights for the prospective line of research

There is evidently a dearth of research into the dynamic development of L2 learners’ affective variables as they grow within different language learning programs. Exploration of this dynamic quality needs to follow from the CDST because of its more realistic and comprehensive view of the complex nature of language learning. Accordingly, there is a further need for CDST compatible research methods, among which the process tracing is one. In SLA domain, this approach has been successfully employed to unravel the causes of boredom and also the challenges of online education. Similarly, other learner related or even teacher-related affective variables can also be explored through process tracing. Instances can include language learners’ motivation, demotivation, grit, foreign language enjoyment and so on. Both positive and negative affective variables can be explored through this method and the implications for the former are that the main sources of positive affective variables will be revealed and can be strengthened. Implications for the latter include the attempts to remove the barriers to negative affects or find ways to compensate for them. The main beneficiaries of such a line of research will be L2 learners and teachers.

Process tracing, which is a qualitative research method, can also be used in mixed approaches if accompanied by a quantitative research method. It is necessary that the other quantitative methods should also be congruent with the CDST line of research, so that they are capable of representing the detailed growing nature of language learners’ affective variables. As suggested by Ford et al. (1989), process tracing can be used to explore neglected research questions, and can also be used together with other qualitative research methods such as protocol analysis in exploring a learning-related variable. In the SLA studies reviewed above, process tracing was used along with interviews to obtain more in-depth data for analysis.

## Conclusive remarks

The dynamicity inherent to L2 learners’ affective variables requires a line of inquiry guided by the CDST. It is necessary to study the processes of change in the affective variables in the reality of classroom learning, as raised by Larsen-Freeman (1997). A comprehensive overview of CDST in applied linguistics (Larsen-Freeman and Cameron, 2008) points to the self-organizing, interconnected and co-adaptive nature of language learning related constructs. Language learners’ affective qualities emerge out of the interactive context of classroom learning. In order to systematically study the processes of change in the development of language learner-related variables including their emotions and bring substantial evidence for that, effective methodological processes are needed, especially those that can reveal the underlying causes of a certain condition. Among the several CDST-compatible qualitative research methodologies, process tracing seems to be a perfect match for exploring language learners’ affective variables, mostly of which have scarcely been investigated dynamically before. Though process tracing has been still less applied in SLA research, in the present study, I brought reasons why it is able to reveal nuances of change in the development of language learners’ affective variables (e.g., their motivation, grit, language learning enjoyment, boredom). Some actual exemplary works were reviewed and their success in revealing the underlying causes of the outcome of interest was proven. There are hopes that prospective SLA literature can effectively use this innovative qualitative research methodology to reveal more about the nuanced development of L2 learners’ affective constructs embedded within the live interactive and ever-changing classroom learning context. Despite the acknowledgment of CDST in the field of SLA, the use of compatible methods with this meta-theory in this field has not been well-established yet. Adopting a CDST perspective, future studies on L2 affective variables can benefit from using case-centric process tracing to systematically generate case-sensitive theories within different situations of L2 learning. Furthermore, L2 affective variables *via* process tracing can be investigated from the lens of positive psychology (for a recent review see Wang et al., 2021) to see their dynamicity in learning and teaching processes. Researchers interested in the situational patterns of L2 affective variables might find the use of process tracing somehow challenging. One challenge might be related to the need for the consideration of alternative explanations of the causal mechanisms underlying the emergence of the variables of interest. For this, L2 researchers should have mastery over the literature of these variables so that they can come up with several alternative interpretations.

## Author contributions

The author confirms being the sole contributor of this work and has approved it for publication.
